# *Akkermansia muciniphila* and Alzheimer’s Disease: Mechanisms, Evidence and Translational Potential

**DOI:** 10.3390/biom16050726

**Published:** 2026-05-15

**Authors:** Jun Li, Qiushuang Long, Binglin Zhu

**Affiliations:** 1 Brain Research Center and State Key Laboratory of Trauma, Burns, and Combined Injury, the Army Medical University (Third Military Medical University), Chongqing 400038, China; lijuncedar@gmail.com; 2 Jinfeng Laboratory, Chongqing 401329, China; longqiushuang@jflab.ac.cn

**Keywords:** *Akkermansia muciniphila*, Alzheimer’s disease, gut–brain axis, postbiotics, microbiome-based therapeutics

## Abstract

*Akkermansia muciniphila* (*A. muciniphila*) is a bacterium that breaks down mucus and is studied for its effects on metabolism and the immune system. Studies show that it affects Alzheimer’s disease (AD) by protecting the gut barrier, reducing inflammation, and influencing communication between the immune system, the brain, and mitochondria. This review summarizes mechanistic, preclinical, and translational evidence connecting *A. muciniphila* to AD, including products such as short-chain fatty acids (SCFAs), and structural or secreted proteins including Amuc_1100 and extracellular vesicles (AmEVs). We also discuss differences between bacterial strains, differences in research methods, and findings that change under different conditions, which make the results harder to interpret. Animal studies suggest neuroprotective effects, but clinical evidence is still limited. Clinical use will need human studies at the strain level, confirmation in humanized models, and early trials using biomarkers to test safety and causal effects.

## 1. Introduction

Alzheimer’s disease (AD) is a major cause of dementia, affecting more than 55 million people worldwide and accounting for approximately 10 million new cases every year [1]. The global prevalence of AD is projected to reach approximately 152 million cases by 2050 [2]. Current therapies, including acetylcholinesterase inhibitors and NMDA receptor antagonists, primarily provide symptomatic relief and have limited impact on disease progression [3,4]. Between 1995 and 2021, more than 140 clinical trials failed to yield approved treatments for AD, despite research investments of about 42.5 billion US dollars [5]. As of early 2025, 182 ongoing clinical trials are evaluating 138 distinct drug candidates targeting a wide range of molecular mechanisms implicated in AD pathogenesis [6]. These findings highlight the biological complexity of AD and that effective treatments to slow or stop the disease are still lacking. Therefore, increasing research studies are focusing on new approaches that consider how factors may contribute to disease development.

Because current therapies have limited efficacy, the gut–brain axis has become an important focus in AD research. The microbiota–gut–brain axis (MGBA) describes bidirectional communication between gut microbes and the brain; however, the specific molecules and pathways involved remain incompletely understood [7,8,9]. Studies in animals and humans show that gut microbes may influence neurodegenerative disease by multiple mechanisms, including the production of microbial metabolites such as short-chain fatty acids (SCFAs), regulation of immune responses, and maintenance of intestinal and blood–brain barrier (BBB) integrity [8,10,11]. In patients with AD, alterations in gut microbiota have been consistently reported, including reduced microbial diversity and compositional shifts. These changes have been associated with systemic inflammation, metabolic dysfunction, amyloid β (Aβ) accumulation, and cognitive decline [12,13,14,15,16].

Because *A. muciniphila* is known for its beneficial effects on metabolism, it has been studied for its potential role in AD. Possible mechanisms include modulation of the gut barrier, immune signaling, and metabolic homeostasis, all of which are processes implicated in AD pathology [17,18,19]. Although its benefits in metabolic diseases are well established (e.g., obesity, type 2 diabetes, and NAFLD) [20,21,22], accumulating evidence indicates that it may also modulate neurodegenerative processes, including AD [17,23,24].

This review focuses on the mechanistic, preclinical, and translational evidence linking *Akkermansia muciniphila* to Alzheimer’s disease. We summarize its roles in gut barrier integrity, immune modulation, metabolic regulation, and gut–brain axis signaling. We also discuss current limitations and future directions for its potential clinical application in AD.

## 2. *A. muciniphila*: Taxonomy, Biology, and Host Interactions

### 2.1. Phylogenetic and Genomic Diversity

*A. muciniphila* is a strictly anaerobic, Gram-negative bacterium that primarily degrades mucin. It belongs to the phylum *Verrucomicrobiota* and represents a distinct lineage. This species is the most widely studied member of the *Akkermansia* genus. Other species in this genus include *A. glycaniphila*, which was isolated from the reticulated python (*Python reticulatus*) [25,26,27]. *A. muciniphila* possesses distinctive genomic features, including an expanded repertoire of mucin-degrading enzymes and specialized metabolic pathways adapted to the mucus niche.

*A. muciniphila* typically accounts for about 1–4% of the gut microbiota in healthy individuals, indicating that it is a common mucin-degrading bacterium in the colon [28]. Genomic comparisons have revealed clear differences among strains of *A. muciniphila*. Based on average nucleotide identity (ANI) and phylogenetic analysis, these strains can be grouped into four major phylogroups, designated AmI to AmIV [29]. The AmI group, including the type strain *MucT* (ATCC BAA-835), can be further subdivided into AmIa and AmIb, which share more than 95% ANI but differ in gene content and metabolic capacity [29]. The AmII group can produce vitamin B12, enabling autonomous propionate production. The AmIII and AmIV groups differ from AmI, with ANI values below 92%, and represent distinct species [29]. However, the functional characteristics of these groups remain incompletely understood. This phylogroup diversity suggests potential functional differences among strains. Recent comparative genomic and strain-resolved analyses further indicate that such heterogeneity may critically influence host-microbe interactions, particularly in metabolic outputs, immune modulation, and ecological adaptation within the mucus niche [30]. Mechanistically, these strain-specific differences are associated with variation in mucin-degrading enzyme repertoires, short-chain fatty acid biosynthetic pathways, and outer membrane-associated signaling molecules, which collectively shape host metabolic and immune responses. However, the extent to which these strain-specific traits translate into differential effects in disease contexts, including AD, remains to be fully elucidated.

### 2.2. Structural and Functional Features

The outer membrane of *A. muciniphila* contains multiple proteins and enzymes involved in mediating host–microbe interactions. Amuc_1100 is a 32 kDa outer membrane protein with a pili-like structure and high stability. It remains active after heat treatment and pasteurization [18,31,32,33,34,35]. *A. muciniphila* can release extracellular vesicles (AmEVs) with a size of about 20–200 nm. These vesicles carry Amuc_1100, lipids, and nucleic acids [36,37,38,39]. In addition to Amuc_1100 and AmEVs, *A. muciniphila* also expresses other outer membrane proteins, such as Amuc_1434, Amuc_2172, and PilQ. It also produces enzymes that facilitate mucin degradation and interaction with the host [40,41,42,43,44,45,46]. These components are critical for the bacterium’s colonization and metabolic function, their direct involvement in AD-related neuropathology has not yet been demonstrated. The specific roles of these structural elements, particularly outer membrane proteins and vesicles, in AD pathophysiology will be further examined in the following section.

### 2.3. Ecological and Metabolic Characteristics

*A. muciniphila* is mainly found at the oxic–anoxic interface of the distal colon. Host-derived mucins serve as the primary carbon and nitrogen source for this bacterium [47]. *A. muciniphila* establishes early in life, reaches relative stability during childhood, and in adults is associated with markers of mucosal health and aging [48]. AmII and AmIV phylogroups display higher microaerotolerance, likely related to enhanced oxidative stress resistance, and are therefore more frequently detected in dysbiotic or inflamed gut environments [48]. *A. muciniphila* has strong mucin-degrading activity and is mainly located in the outer mucus layer. It is associated with increased mucus thickness, enhanced goblet cell activity, and improved epithelial barrier function [49,50].

By fermenting mucoprotein, *A. muciniphila* generates SCFAs, primarily acetate, propionate, and butyrate [51,52]. These metabolites can act on host cells through G-protein-coupled receptors (e.g., GPR41 and GPR43) and epigenetic modulation via histone deacetylase inhibition, thereby influencing immune cell function and epithelial barrier integrity [53]. Strains within the AmII and AmIV phylogroups are capable of synthesizing vitamin B12 de novo, thereby enabling propionate production via the methylmalonyl-CoA pathway [53].

Taken together, the localization of *A. muciniphila* within the mucus layer, its capacity to produce SCFAs, and the immunomodulatory outer membrane components such as Amuc_1100 are closed with mucosal homeostasis in the human gut. These ecological and metabolic features are consistent with reported effects of *A. muciniphila* on intestinal barrier function, inflammation, and gut–brain axis signaling in the context of AD.

## 3. Mechanistic Pathways Linking *A. muciniphila* to AD

*A. muciniphila* has been linked to multiple processes relevant to AD, including intestinal barrier integrity, neuroinflammation, metabolic–neural signaling, and mitochondrial homeostasis. These effects are mediated through many bioactive components, such as SCFAs, Amuc_1100, and AmEVs.

Recent studies indicate that the functional potential of *A. muciniphila* is strain-dependent. Strains within the AmI phylogroup exhibit enhanced SCFAs production, improved adherence to the mucosal surface, increased oxygen tolerance, and more potent immunomodulatory activity, including the promotion of regulatory T cell (Treg) differentiation and elevated IL-10 expression. Transcriptomic data show that AmI strains induce broad host transcriptional changes, consistent with potential effects on gut–brain axis signaling [29]. These findings highlight strain-level differences as an important source of functional variability in *A. muciniphila*, with implications for both mechanistic studies and therapeutic development in AD.

### 3.1. Barrier Integrity and Gut–Brain Axis Protection

In AD, impairment of the intestinal barrier and BBB allows microbial components and pro-inflammatory mediators to enter the circulation and the central nervous system, which is associated with neuroinflammation and cognitive decline [24,54]. *A. muciniphila* has been reported to influence this process through several mechanisms, including SCFAs and outer membrane proteins.

**Figure 1:**
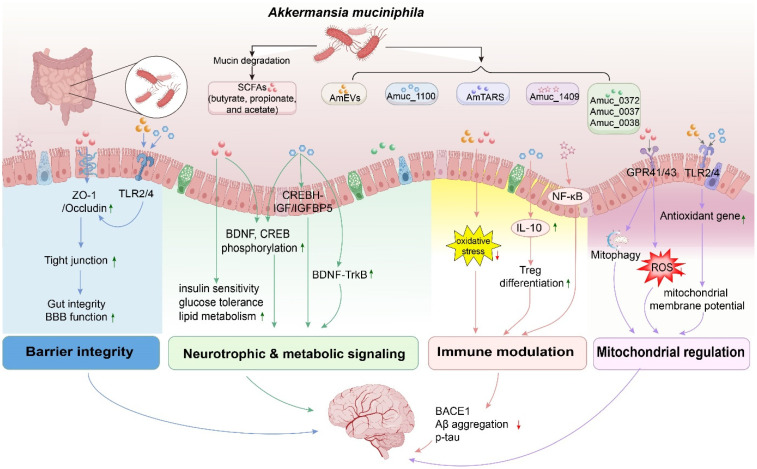
Schematic overview of the neuroprotective mechanisms of *A. muciniphila* in Alzheimer’s disease (AD). *A. muciniphila* and its derivatives, including short-chain fatty acids (SCFAs), outer membrane proteins such as Amuc_1100 and Amuc_1409, and extracellular vesicles (AmEVs), contribute to four major functional modules: barrier integrity, neurotrophic and metabolic signaling, immune modulation, and mitochondrial regulation. These effects involve multiple signaling pathways, including GPR41/43, TLR2/4, NF-κB, IL-10, and CREB-BDNF-TrkB. Through these mechanisms, *A. muciniphila* enhances gut– and blood–brain barrier (BBB) function, promotes synaptic plasticity, suppresses systemic and neuroinflammation, and improves mitochondrial function and redox balance. As a result, metabolic homeostasis is maintained, Aβ and tau pathology are attenuated, and cognitive performance is improved, highlighting the role of *A. muciniphila* as a modulatory factor in the microbiota–gut–brain axis in AD (this figure was created using Adobe Illustrator v30.0 (2026)). Arrows in different colors (blue, green, red, purple) are used to distinguish between different functional pathways (e.g., green for Neurotrophic & metabolic signaling, red for Immune modulation, etc.).

SCFAs play distinct roles in gut–brain communication. Butyrate can improve intestinal barrier integrity, and influence Aβ and tau pathology [55]. Propionate helps maintain mitochondrial function and supports homeostasis in both the gut and brain [56]. Acetate can strengthen the mucosal barrier and reduce neuroinflammation. It does this by increasing SCFA levels, limiting excessive microglial synaptic pruning, and protecting hippocampal synapses under stress conditions such as sleep deprivation [57]. In animal models of AD, treatment with *A. muciniphila* reduces gut permeability, decreases Aβ accumulation in the brain, and improves metabolic imbalance [58,59]. Polysaccharide-based methods that increase *A. muciniphila* levels, such as PSP-1, can help restore gut–brain barrier balance and improve cognitive performance [60]. These findings support the potential use of *A. muciniphila* as an adjunct therapeutic strategy (see Figure 1, barrier integrity module).

### 3.2. Immunomodulation and Neuroinflammation Suppression

Chronic neuroinflammation, driven by microglial activation and systemic immune imbalance, is a hallmark of AD [61,62]. Evidence indicates that *A. muciniphila* reduces systemic lipopolysaccharide (LPS) levels and pro-inflammatory mediators, which is associated with decreased cortical amyloid-β deposition and improved memory performance in experimental models [24].

Preclinical studies indicate that butyrate functions as a histone deacetylase (HDAC) inhibitor [63], thereby modulating gene expression programs linked to synaptic plasticity and cognition [64]. In AD mouse models, butyrate has been reported to limit oxidative stress and reduce amyloid-β-induced neuronal toxicity via the GPR109A-mediated regulation of APP, NEP, and BDNF expression [65]. In 5xFAD mice, butyrate treatment is associated with a lower cerebral amyloid-β burden, improved learning and memory performance [66], and reduced tau hyperphosphorylation and glial activation, partly through changes in inflammatory gene expression [67]. Amuc_1100, a major outer membrane protein of *A. muciniphila*, exerts immunomodulatory effects primarily through TLR2 and TLR4 signaling. It induces IL-10 production, enhances epithelial barrier function, and reduces pro-inflammatory cytokine expression. Although these findings support its anti-inflammatory and homeostatic roles, potential links to regulatory T cell differentiation and the AhR-kynurenine pathway in AD remain incompletely defined and require additional experimental validation [31,68,69,70,71,72]. AmEVs suppress MAPK signaling and downregulate pro-inflammatory cytokines, thereby reducing the inflammatory burden [73].

In vivo studies indicate that supplementation with *A. muciniphila* reduces hippocampal levels of TNF-α, IL-1β, IL-6, and IFN-γ, leading to improved cognitive performance in tauopathy and AD mouse models [74]. Clinical observations indicate that *A. muciniphila* abundance is negatively associated with cerebral amyloid burden in patients with mild cognitive impairment [75]. These findings highlight the relevance of strain-specific effects and suggest that strain selection should be carefully considered in therapeutic applications [70,76,77] (see Figure 1, immune modulation module).

### 3.3. Metabolic-Neural Crosstalk

Systemic metabolic changes are commonly observed in AD, and growing evidence links gut microbiota activity to brain function through the gut–brain axis [78,79]. Within this context, *A. muciniphila* contributes to this metabolic–neural crosstalk through SCFAs, the outer membrane protein Amuc_1100, and extracellular vesicles, which contribute to pathways relevant to neuronal and cognitive processes.

#### 3.3.1. Metabolic Modulation

SCFAs, particularly propionate and butyrate, are associated with improved systemic metabolic control, including glucose homeostasis, insulin sensitivity, and lipid metabolism, in part through effects on enteroendocrine signaling and GLP-1 secretion [80,81,82]. SCFAs have also been shown to stimulate intestinal gluconeogenesis and incretin release, which are linked to satiety and improved energy balance [83]. These systemic changes may contribute to reduced neuroinflammation, enhanced cerebral energy utilization, and the maintenance of cerebrovascular integrity in AD. In mouse models of AD, supplementation with *A. muciniphila* improved glucose tolerance, strengthened intestinal barrier function, and reduced lipid metabolic disturbances. These improvements are also associated with lower levels of Aβ40/42 and better performance in spatial learning and memory tasks [59]. Together, these metabolic changes are associated with improved regulation of neurotransmission and neurotrophic signaling, likely through the maintenance of energy balance and cellular homeostasis, thereby linking systemic metabolic status to neuronal stability and cognitive performance.

#### 3.3.2. Neurotransmitter and Neurotrophic Modulation

In addition to their roles in barrier reinforcement and immune regulation, SCFAs and Amuc_1100 have been implicated in the central neurotransmission and neurotrophic signaling. Through the release of microbial metabolites, bioactive proteins, and extracellular vesicles, *A. muciniphila* may influence multiple neurotransmitter-related pathways, including γ-aminobutyric acid (GABA), serotonin (5-HT), dopamine, and brain-derived neurotrophic factor (BDNF).

GABA: GABA is the principal inhibitory neurotransmitter in the central nervous system and plays an essential role in maintaining excitatory–inhibitory balance. Genomic analyses have identified putative GABA-related metabolic genes in certain *A. muciniphila* strains [84]. However, current evidence linking *A. muciniphila*-derived GABA to AD-related pathology remains limited and largely indirect. Although alterations in gut microbiota composition, including GABA-producing bacteria, have been associated with changes in peripheral GABA levels, it remains unclear whether *A. muciniphila* directly modulates central GABAergic signaling or contributes to AD progression through this pathway [85,86]. Therefore, GABA-related effects should be regarded as a potential but not yet established mechanism in *A. muciniphila*-mediated gut–brain interactions, warranting further targeted investigation.

Serotonin and BDNF: Serotonin and brain-derived neurotrophic factor (BDNF) may also be influenced by *A. muciniphila*. The bacterium enhances serotonergic signaling by upregulating tryptophan hydroxylase 1 (TPH1) and downregulating indoleamine 2,3-dioxygenase 1 (IDO1), thereby promoting serotonin biosynthesis from tryptophan while reducing flux through the kynurenine pathway [87]. Both *A. muciniphila* and its extracellular vesicles have been reported to increase serotonin levels and regulate the expression of serotonin-related genes in the colon and the hippocampus. These observations suggest a potential role in serotonin signaling along the gut–brain axis [88].

Animal studies indicate that *A. muciniphila* and Amuc_1100 can increase serotonin production in the gut by upregulating TPH1 expression and reducing serotonin transporter (SERT) levels. As a result, peripheral serotonin levels increase and can also be detected in the prefrontal cortex. These changes are accompanied by improvements in mood-related behaviors [89,90,91]. Administration of *A. muciniphila* increases BDNF expression in the hippocampus and enhances serotonin-related signaling in gut–brain axis models [92]. Amuc_1100 has been shown to restore BDNF/TrkB signaling in the hippocampus and cortex in antibiotic-induced depressive mice models, leading to reduced anxiety- and depression-like behaviors [93]. Similarly, supplementation with *A. muciniphila* increases BDNF activity and improves spatial memory in mouse models [94]. Together, these results suggest a potential serotonin-BDNF pathway through which *A. muciniphila* may support synaptic plasticity, learning and memory.

Dopamine: Preclinical studies suggest that *A. muciniphila* may affect dopamine-related pathways, but its role in AD remains unclear. In Parkinson’s disease (PD) models, supplementation with *A. muciniphila* increases butyrate production and protects dopaminergic neurons by reducing microglial activation [95]. However, antibiotic-induced gut imbalance leads to overgrowth of *A. muciniphila*, which is accompanied by lower dopamine levels and increased systemic inflammation [96]. Studies on the liver–brain axis indicate that the higher levels of *A. muciniphila* are associated with cognitive improvement, mainly linked to serotonin and BDNF signaling, whereas dopamine-related pathways appear to play a lesser role [97]. Together, these findings suggest that the interaction between *A. muciniphila* and dopamine signaling depends on the context. They also indicate that results from PD models cannot be directly applied to AD, where direct evidence is still limited (see Figure 1, neurotrophic & metabolic signaling module).

### 3.4. Cellular Homeostasis: Oxidative Stress and Mitochondrial Regulation

Oxidative stress and mitochondrial dysfunction are hallmarks of AD [98,99]. Studies from metabolic and inflammatory disease models indicate that *A. muciniphila* can reduce oxidative damage and protect mitochondrial function [100,101,102,103,104]. Although direct evidence in AD remains limited, preclinical studies suggest that *A. muciniphila* and its metabolites may regulate mitochondrial homeostasis through mechanisms related to AD pathology [14,56,105]. Consistent with these findings, higher levels of *A. muciniphila* are associated with better cognitive performance and lower oxidative stress in models of mild cognitive impairment and AD [14,105].

SCFAs, especially propionate, play an important role in these protective effects. In mouse models of AD, propionate improves cognitive performance and maintains mitochondrial homeostasis. This effect is linked to reduced mitochondrial fission and increased mitophagy, involving DRP1, PINK1/PARKIN, and GPR41/43 signaling [56]. Butyrate supports neuronal resistance by improving mitochondrial function. Sodium butyrate has been reported to increase mitochondrial respiration, enhance antioxidant defenses such as superoxide dismutase (SOD1), and reduce reactive oxygen species (ROS) levels via NRF2- and GPR109A-mediated signaling. These effects protect neurons from oxidative stress [65,106,107]. In contrast, evidence for other components derived from *A. muciniphila* remains limited and is largely derived from non-AD models. AmEVs are associated with reduced oxidative markers and improved barrier function in inflammatory models [73]. Although these findings provide insight into mechanisms relevant to AD, direct evidence from AD-specific models remains limited (see Figure 1, mitochondrial regulation module).

These mechanisms are summarized in Figure 1, which illustrates the potential pathways through which *A. muciniphila* and its derivatives may influence AD-related processes.

## 4. Translational Evidence and Cognitive Outcomes

Recent translational studies indicate that *A. muciniphila* is involved in AD pathology. However, findings are not consistent and vary across experimental models, disease stages, and human populations.

### 4.1. Alterations in AD Models and Human Studies

Preclinical studies in animal models of AD indicate that the composition of the gut microbiota, including levels of *A. muciniphila*, undergo dynamic changes during disease progression (Table 1). In transgenic mouse models such as APP/PS1 and 3 × Tg-AD, researchers have observed shifts in *A. muciniphila* abundance concurrent with the accumulation of amyloid-β (Aβ) and the manifestation of cognitive deficits. A seminal study by Harach et al. (2017) [108] using germ-free APP transgenic mice demonstrated that the absence of gut microbiota significantly reduced cerebral amyloid pathology. Conversely, transplanting microbiota from conventional APP mice into these germ-free animals restored amyloid deposition. Further supporting a potential protective role, research showed that supplementing with *A. muciniphila* reduced hippocampal plaque load and improved recognition memory in an AD mouse model [59]. Interestingly, sequencing analyses have revealed that *A. muciniphila* levels can increase during presymptomatic stages (1–3 months of age), even before significant amyloid deposition becomes evident [109]. This temporal fluctuation suggests that changes in *A. muciniphila* may represent an adaptive or compensatory response in the early phases of the disease, rather than serving as a consistent biomarker of established pathology.

Human studies are more heterogeneous (Table 2). Several investigations in Chinese, Egyptian, and European cohorts reported higher fecal levels of *A. muciniphila* in patients with AD or mild cognitive impairment (MCI) compared with healthy controls [12,110,111,112]. These increases were also observed in mild AD and early MCI, suggesting that alterations in *A. muciniphila* may occur during early disease stages, such as MCI [113]. In contrast, other studies reported lower levels of *A. muciniphila* in AD or MCI patients, or found no significant difference compared with controls [56]. Such variability is likely influenced by multiple factors. Disease stage appears to be important, as *A. muciniphila* levels may increase in early stages but decline as the disease progresses and systemic inflammation worsens. Metabolic conditions such as obesity and diabetes can affect *A. muciniphila* level and complicate its association with AD-related pathology [14,75]. Hosts’ genetic background may also play a role, particularly APOE ε4 status, which is linked to both AD risk and gut microbiota composition. Moreover, variation in experimental methods and population characteristics, including diet and geographic background, likely contributes to contribute to the differences observed between studies [114]. Better comparability may be achieved through the use of stratified cohorts and standardized sampling and analytical methods. Consideration of diet and genetic background may further improve interpretation. Finally, approaches beyond 16S rRNA sequencing, including metagenomics and metabolomics, could help clarify the relationship between *A. muciniphila* and AD.

**Table 1:** Changes in *A. muciniphila* in Alzheimer’s disease animal models.

Experimental Subjects	Intervention	Changes in the Abundance of *A. muciniphila* in AD Models	*A. muciniphila* Intervention and Its Effect	References
WT + PBS (*n* = 6) WT + AKK (*n* = 6) APP/PS1 + PBS (*n* = 10) APP/PS1-HFD (*n* = 10) APP/PS1 + AKK (*n* = 10) APP/PS1-HFD + AKK (*n* = 10)	real-time PCR	Decreased	*A. muciniphila* (5 × 10^9^ CFU/0.2 mL) treatment: - Reduction in fasting blood glucose - Improvement in glucose tolerance - Restoration of intestinal barrier - Normalization of lipid metabolism - Reduction in hippocampal Aβ plaques - Decrease in cortical soluble Aβ40 and Aβ42 - Improvement in cognitive and behavioral performance	[59]
C57BL/6 WT (*n* = 7) APPPS1 (*n* = 6)	16S rRNA sequencing	Decreased	NA	[108]
WT mice at 1, 2, 6, and 9 months, *n* = 14, 17, 31, and 18, respectively; APP/PS mice at 1, 2, 6, and 9 months, *n* = 21, 24, 34, and 18, respectively	16S rRNA sequencing	Increased	NA	[109]
Control (CG) (*n* = 100) T2DM (*n* = 100) T2DM-AD (TA) (*n* = 100) T2DM-AD + AKK treatment (TAAT) (*n* = 100) T2DM-AD + AKK prevention (TAAP) (*n* = 100)	16S rRNA sequencing	Decreased	Pasteurized *A. muciniphila* (5 × 10^9^ CFU/10 µL) treatment: - Decreased *Proteobacteria* - Increased behavioral performance - Reduced β-amyloid (Aβ) levels - Decreased tau protein levels - Reduced IFN-γ levels - Increased IL-4 and IL-1 levels	[115]
Control (*n* = 4) AlCl3-induced rats (*n* = 4) AlCl3-induced rats + AKK (*n* = 5)	16S rRNA sequencing	Decreased	*A. muciniphila* (1 × 10^9^ CFU/200 µL) treatment: - Increased beneficial bacteria - Decreased harmful bacteria - Improved cognitive function - Reduced Aβ levels - Decreased phosphorylated tau (p-tau) proteins	[58]
Sham (*n* = 10) Sham + AKK (*n* = 10) APP/PS1 (*n* = 10) APP/PS1 + AKK (*n* = 10)	NA	Decreased	*A. muciniphila* (1 × 10^9^ CFU/200 µL) treatment: - Lowered pro-inflammatory markers - Enhanced memory performance - Reduced anxiety behavior	[74]
C57BL/6JRj WT (*n* = 8) APP/PS1 (*n* = 7) APP/PS1 + AKK (*n* = 7) APP/PS1 + AKK + GOS (*n* = 8)	16S rRNA sequencing	Decreased	*A. muciniphila* (1 × 10^9^ CFU/100 µL) and GOS treatment: - Increased gut microbiota α-diversity - Restoration of fasting glucose and glucose metabolism to wild-type levels - Reduced anxiety-like behavior - Improved long-term and short-term memory - Decreased soluble Aβ_1-42_ levels - Reduced microglial activation - Attenuated neuroinflammation	[116]
C57BL/6J WT (*n* = 9) APP/PS1 (*n* = 7) APP/PS1 + capsaicin (*n* = 7)	16S rRNA sequencing	Increased	NA	[117]
C57BL/6J WT (*n* = 10) 5xFAD (*n* = 5) AKK_5xFAD (*n* = 4)	16S rRNA sequencing	Unchanged	*A. muciniphila* (2 × 10^9^ CFU/mL) treatment: - Increased microgliosis - Unchanged Aβ plaque burden - Unaltered astrogliosis	[118]

AD, Alzheimer’s disease; AKK, *A. muciniphila*; CFU, colony-forming unit; GOS, galactooligosaccharides; HFD, high-fat diet; NA: not available; T2DM, type 2 diabetes mellitus.

**Table 2:** Alterations of *A. muciniphila* in Alzheimer’s disease patients.

Experimental Subjects	Analysis	Changes in the Abundance of *A. muciniphila* in Patients	*A. muciniphila* Intervention and Its Effect	References
AD patients (*n* = 25) Healthy controls (*n* = 25)	16S rRNA sequencing	Increased	NA	[111]
AD patients (*n* = 13) Healthy controls (*n* = 13)	16S rRNA sequencing	Increased	NA	[110]
AD patients (*n* = 100) Healthy controls (*n* = 71)	16S rRNA sequencing	Increased	NA	[114]
AD patients (*n* = 19) Healthy controls (*n* = 18)	16S rRNA sequencing	Decreased	*A. muciniphila* treatment (2 × 10^8^ CFU/mL) of 5× FAD mice: - Upregulated GPR41 and GPR43 signaling pathways - Enhanced DRP1-mediated mitochondrial fission and PINK1/PARKIN-dependent mitophagy - Improved mitochondrial homeostasis - Enhanced cognitive function - Increased mitochondrial dynamics	[56]
MCI (*n* = 30) Controls (*n* = 30)	16S rRNA sequencing	Decreased	NA	[14]
MCI (*n* = 119) Normal controls (*n* = 320)	Shotgun metagenomic sequencing	Decreased	NA	[75]
AD patients (*n* = 30) MCI patients (*n* = 30) Normal controls (*n* = 30)	16S rRNA sequencing	Increased	NA	[113]
AD patients (*n* = 581) Healthy controls (*n* = 445)	16S rRNA sequencing	Increased	NA	[12]
GDS-3 (MCI) (*n* = 4) GDS-4 (mild AD) (*n* = 4) GDS-5 (moderate AD) (*n* = 4) Healthy controls (*n* = 10)	16S rRNA sequencing	Increased	NA	[112]

AD, Alzheimer’s disease; CFU, colony-forming unit; MCI, mildly cognitive impaired; NA: not available.

### 4.2. Safety, Formulation, and Translational Considerations

Before *A. muciniphila* can be used clinically, its safety must be established. A randomized placebo-controlled trial in overweight, insulin-resistant adults demonstrated that both live and pasteurized *A. muciniphila* were safe and well-tolerated. The pasteurized form improved insulin sensitivity, reduced plasma cholesterol and inflammatory markers, with no adverse events [119]. Genomic and phenotypic analyses revealed no transmissible antibiotic-resistance genes, supporting its classification as a next-generation probiotic [120]. Pasteurization preserved the activity of outer-membrane proteins such as Amuc_1100, indicating feasibility for postbiotic formulations [119]. These data provide an initial basis for clinical translation, though trials in cognitively impaired populations are still lacking.

Preclinical studies suggest that both live and pasteurized *A. muciniphila* exert neuroprotective effects. In a zebrafish model of type-2 diabetes with AD-like features, pasteurized preparations improved glycemic control, behavior, and memory [115]. In diabetic db/db mice with cognitive impairment, live bacteria provided greater protection of synaptic integrity, while both forms reduced neuroinflammation and modulated gut microbiota and metabolic profiles, implicating microbiota–metabolite–brain interactions [121]. Thus, both viable and non-viable formulations may have therapeutic value; pasteurized bacteria appear more translatable, whereas live preparations show stronger effects on synaptic integrity in preclinical models.

Despite these positive results, several practical issues continue to limit large-scale application. Producing *A. muciniphila* in large quantities is challenging, as the bacterium requires strictly anaerobic conditions and is highly sensitive to oxygen. Another practical concern is whether the bacteria can remain viable during storage and after gastrointestinal transit. To address these limitations, various delivery strategies have been explored. For example, gastric-protective microencapsulation methods, such as calcium–alginate hydrogel matrices, can improve bacterial stability during refrigerated storage and under simulated gastrointestinal conditions [122,123]. Thermostable lyophilized postbiotics are also being considered. These non-viable preparations can retain biological activity, as demonstrated in other probiotic systems [124], and may offer advantages for administration and storage stability.

Further questions remain regarding dosing, treatment duration, and individual host factors, including age, APOE genotype, and baseline gut microbiota composition. Biomarker-guided intervention studies will help determine whether metabolic and immunomodulatory benefits are associated with cognitive protection in AD. Although preclinical studies provide compelling neuroprotective evidence, interspecies differences in immune architecture, gut microbial ecology, and neurodevelopmental dynamics limit direct extrapolation from animal models to humans. These limitations highlight the need for humanized models and patient-centered clinical trials to better evaluate the clinical potential of *A. muciniphila* in AD.

### 4.3. Translational Roadmap

Although preclinical and early translational findings support the therapeutic potential of *A. muciniphila*, a clear stepwise translational pathway is still required. This pathway should include several key components.

First, identification and functional characterization of strain-specific properties are essential, as accumulating evidence indicates substantial heterogeneity across phylogroups in metabolic activity and immunomodulatory effects. Second, the development of stable and clinically applicable formulations, including pasteurized bacteria, postbiotics, and microencapsulation strategies, is necessary to improve safety, scalability, and delivery efficiency. Third, biomarker-guided early-phase clinical trials should be implemented, integrating cognitive assessments with neuroimaging, metabolomic profiling, and inflammatory markers to establish causality and therapeutic relevance. Finally, given the multifactorial nature of AD, combination strategies incorporating *A. muciniphila* with pharmacological agents (e.g., GLP-1 receptor agonists), dietary interventions, or lifestyle modifications may enhance therapeutic efficacy.

Collectively, this framework provides a structured basis for bridging experimental findings and clinical application, and may facilitate the rational design of microbiota-based interventions for AD.

## 5. Limitations and Controversies

### 5.1. Context-Dependent Pro-Inflammatory Potential of A. muciniphila

Although *A. muciniphila* is well known for its metabolic and immunomodulatory benefits, some evidences suggest that its effects may be deleterious under certain inflammatory or immunodeficient conditions. In a dextran sodium sulfate (DSS)-induced colitis model using *Il10*^-/-^ mice, Seregin et al. (2017) [76] reported that colonization with *A. muciniphila* worsened colonic inflammation by increasing microbial translocation and disrupting epithelial barrier repair, an effect linked to impaired NLRP6 inflammasome signaling. Similarly, Ganesh et al. (2013) [77] reported that in gnotobiotic mice infected with *Salmonella Typhimurium*, the presence of *A. muciniphila* was associated with enhanced intestinal inflammation and increased systemic immune activation. However, subsequent studies have demonstrated that *A. muciniphila* can also exert protective effects against enteric infection, underscoring its context-dependent role in host–microbe interactions [125]. These findings indicate that the effects of *A. muciniphila* are highly dependent on the host immune status and the surrounding microbial environment.

Beneficial effects of *A. muciniphila* are more frequently reported in hosts with preserved immune and barrier function, whereas different responses have been observed in settings of immune or barrier impairment. In inflammatory and aging-related conditions such as AD, host responses may differ from those observed in healthy populations. Host-related factors, including APOE genotype, inflammatory status, disease stage, and strain differences, are therefore important considerations in therapeutic applications.

### 5.2. Inconsistencies in Preclinical and Clinical Findings

Neuroprotective effects of *A. muciniphila* have been observed in several studies, but results vary across model systems. In some transgenic mouse models of AD, supplementation with *A. muciniphila* did not reduce Aβ levels. In other cases, increased microglial activation was observed without clear changes in plaque burden.

Similar variability has also been observed in human studies. Across different cohorts, studies report inconsistent changes in abundance of *A. muciniphila* in patients with AD or MCI. These differences occur across studies that vary in design, sampling strategy, and cohort characteristics. Hosts’ genetic background, disease stage, and gut microbial composition differ between cohorts, and strain-level variation, together with study design, limit clear interpretation. Without adequate experimental control and appropriate population stratification, the role of *A. muciniphila* in disease progression and its therapeutic relevance remains unclear.

### 5.3. Methodological and Translational Barriers

Comparison across studies is limited. Different methods are used, including 16S rRNA sequencing and metagenomic analysis, and samples are obtained from various sources such as feces, mucosa, and plasma. These methodological differences are associated with inconsistent findings. Study populations also vary between reports. Diet, disease status, and APOE genotype differ across cohorts and are not always well controlled, making it difficult to identify microbiome features consistently associated with AD. Differences in sample processing and data analysis further limit comparability across studies.

Clinical data remain scarce. Some studies include small sample sizes, short intervention periods, or observational designs. Additionally, strain-level information is often unavailable, making it difficult to assess whether findings from animal models can be translated to humans. Studies with longer follow-up and improved population stratification may enhance future research. The use of multi-omics approaches, such as metagenomics and metabolomics, may help improve understanding of interactions among *A. muciniphila*, the gut–brain axis, and AD.

## 6. Future Perspectives

*A. muciniphila* has been studied primarily for its involvement in the gut–brain axis and its reported effects on metabolism, immune responses, and neuroprotection in AD. Translating findings from experimental studies into clinical applications will require input from multiple research disciplines to address existing limitations. Computational tools, including artificial intelligence-based methods, may be useful for examining host-microbe interactions and for guiding more targeted intervention strategies.

### 6.1. Clinical Validation and Precision Stratification

Current clinical evidence linking *A. muciniphila* to AD and cognitive outcomes remains limited. Most human data are derived from short-term dietary interventions or observational studies, including the modified Mediterranean ketogenic diet (MMKD) in patients with mild cognitive impairment (MCI) [85]. Systematic reviews indicate that although *A. muciniphila* shows potential benefits in metabolic and inflammatory diseases, clinical trials specifically focused on cognitive decline or AD remain scarce. Existing trials also vary widely in study design, dose, treatment approach, and patient stratification [126,127,128]. Future clinical trials should focus on patients with early-stage or biomarker-confirmed AD and incorporate multiple outcome measures. These may include cognitive assessments, advanced brain imaging, and cerebrospinal fluid (CSF) biomarkers. Stratifying patients based on APOE genotype and baseline gut microbiota composition may help identify subgroups that respond more favorably to treatment.

Validated biomarkers are also important for monitoring treatment response. In addition to cognitive assessments, biomarkers may include strain-level features of *A. muciniphila*, inflammatory markers in cerebrospinal fluid (CSF) and blood, and metabolomic profiles of SCFAs and related metabolites. Brain imaging techniques, such as amyloid- and tau-PET and resting-state fMRI, may also be applied. Integrating these biomarkers with host genetic markers, such as APOE and TREM2, may enable more precise stratification of AD patients for *A. muciniphila*-based interventions.

### 6.2. Mechanistic Exploration and Engineering Strategies

Several candidate pathways have been described, including SCFA-GPCR signaling that reduces microglial activation and synaptic loss [56], as well as TLR2-Amuc_1100 signaling linked to increased serotonin and BDNF levels and reduced neuroinflammation [91]. However, neuroimmune mechanisms specific to AD remain poorly characterized, particularly at strain-level resolution. Advanced tools, such as CRISPR-based microbial editing, spatial transcriptomics across gut–brain axis tissues, and iPSC-derived BBB organoids, may help establish causal links and reveal previously unrecognized signaling pathways.

Therapeutic delivery remains a challenge. Current developments include gastric-protective microencapsulation to support probiotic survival [122], as well as thermostable lyophilized postbiotics that retain bioactivity and enable non-oral administration [129]. Genetic engineering also broadens therapeutic possibilities. For example, engineered *A. muciniphila* strains could be designed to secrete anti-amyloid peptides, deliver inflammation-responsive nanobodies, or enhance the production of neuroprotective metabolites such as indoles and polyamines [130]. These approaches support the use of *A. muciniphila* as a next-generation living biotherapeutic platform.

### 6.3. Combination Therapies and Translational Challenges

Because AD involves multiple pathogenic processes, *A. muciniphila*-based monotherapy is unlikely to be sufficient on its own. Preclinical and early clinical studies suggest synergistic benefits when combined with GLP-1 receptor agonists such as liraglutide and semaglutide, which have been shown to reduce amyloid-β deposition, tau hyperphosphorylation, and microglial activation [131,132]. Lifestyle interventions, such as Mediterranean or ketogenic diets, aerobic exercise, and cognitive training, can complement microbial approaches and are associated with changes in neuroplasticity, microbial diversity, and neuroinflammation status [133,134,135].

Several translational challenges remain, including small and heterogeneous study cohorts, inconsistent microbial strain characterization, limited biomarker validation, and insufficient consideration of genetic risk factors. In future studies, several aspects should be addressed. First, engineered *A. muciniphila* strains may be developed to enhance the production of neuroactive metabolites. Second, there is a need to standardize formulations that meet GMP requirements, such as lyophilized or enteric-coated products. Third, combination therapies should be further evaluated, including the use of *A. muciniphila* together with GLP-1 agonists, polyphenols, or lifestyle interventions. Finally, multi-omics approaches, including metagenomics, metabolomics, and immune profiling, could support the development of more personalized treatment strategies.

### 6.4. Regulatory and Ethical Considerations

The use of *A. muciniphila* requires careful consideration of strain differences and safety concerns. Different phylogroups of *A. muciniphila* (AmI-AmIV) exhibit distinct effects on immune function, metabolism, and neuroactive pathways. However, most clinical studies do not analyze bacteria at the strain level, which limits the reproducibility of results. The use of engineered strains also raises safety concerns, including potential gene transfer, prolonged persistence in the gut, and unintended interactions with other gut microbes. Regulatory agencies therefore require appropriate frameworks to evaluate long-term safety, persistence in the gut, and potential adverse effects of live bacterial therapies. This is particularly important for patients with AD, who often exhibit compromised immune function and impaired gut barrier integrity.

## 7. Conclusions and Outlook

Based on current evidence, *A. muciniphila* may be associated with AD, but its effects appear to be context-dependent. Animal studies indicate that *A. muciniphila* can influence immune function, metabolism, and gut–brain interactions, with potentially beneficial effects under certain conditions. Human studies suggest that *A. muciniphila* is generally safe and may confer metabolic benefits, supporting further clinical investigation. However, clinical findings remain inconsistent across studies, likely due to differences in study designs, population characteristics, and the lack of strain-level analysis. Practical challenges, including difficulties in large-scale cultivation, formulation, and maintaining bacterial viability in the gut, also limit clinical application.

Large-scale studies with longer follow-up periods and more standardized methodologies are still needed. Integrating multi-omics data with well-defined clinical outcomes and appropriate patient stratification may improve understanding of this field. It is also important to study *A. muciniphila* at the strain level to determine whether live bacteria, pasteurized products, or postbiotics are more suitable in terms of safety and efficacy. Overall, further studies are required to clarify how *A. muciniphila* functions at different stages of AD and across diverse host conditions. At present, it is premature to consider it a reliable treatment option for AD.

## Data Availability

No new data were generated or analyzed in this study. All data discussed in this review are available from the cited literature and publicly accessible databases as referenced in the text.

## Abbreviations

Aβ	Amyloid β
AD	Alzheimer’s disease
ADP	Adenosine diphosphate
*A. muciniphila*	*Akkermansia muciniphila*
AmEVs	*Akkermansia muciniphila*-derived extracellular vesicles
ALPK1	Alpha kinase 1
ALT	Alanine aminotransferase
ANI	Average nucleotide identity
APP	Amyloid precursor protein
ATCC	American Type Culture Collection
BBB	Blood–brain barrier
BDNF	Brain-derived neurotrophic factor
CREBH	Cyclic AMP-responsive element-binding protein H
CSF	Cerebrospinal fluid
DHA	Docosahexaenoic acid
DNA	Deoxyribonucleic acid
DRP1	Dynamin-related protein 1
DSS	Dextran sodium sulfate
EVs	Extracellular vesicles
GABA	γ-Aminobutyric acid
GFAP	Glial fibrillary acidic protein
GLP-1	Glucagon-like peptide-1
GPR41/43	G-protein-coupled receptors 41 and 43
HDAC	Histone deacetylase
IDO1	Indoleamine 2,3-dioxygenase 1
IFN-γ	Interferon-γ
IL-1β/IL-6/IL-10	Interleukin-1β/6/10
LPS	Lipopolysaccharide
MAPK	Mitogen-activated protein kinase
MCI	Mild cognitive impairment
MMKD	Modified Mediterranean ketogenic diet
MPS	Manuscript processing system
MRI	Magnetic resonance imaging
NF-κB	Nuclear factor kappa B
NMDA	N-methyl-D-aspartate
NLRP6	NOD-like receptor family pyrin domain containing 6
NRF2	Nuclear factor erythroid 2-related factor 2
PET	Positron emission tomography
PSP-1	Polysaccharopeptide-1
PINK1	PTEN-induced kinase 1
ROS	Reactive oxygen species
SCFAs	Short-chain fatty acids
SERT	Serotonin transporter
SOD1	Superoxide dismutase 1
TLR2/TLR4	Toll-like receptor 2/4
TPH1	Tryptophan hydroxylase 1
TrkB	Tropomyosin receptor kinase B
ZO-1	Zonula occludens-1
